# Advancements in Restless Leg Syndrome Management: A Review of Physiotherapeutic Modalities and Their Efficacy

**DOI:** 10.7759/cureus.46779

**Published:** 2023-10-10

**Authors:** Grisha Ratnani, Pallavi Harjpal

**Affiliations:** 1 Neuro-Physiotherapy, Ravi Nair Physiotherapy College, Datta Meghe Institute of Higher Education and Research (DU), Wardha, IND

**Keywords:** rehabilitation, physiotherapy, stretching, physical therapy, exercise, restless legs syndrome

## Abstract

The goal of this review is to compile information on the use of physiotherapeutic treatments for the treatment of restless leg syndrome (RLS) and to classify the effectiveness of conservative methods in relieving the discomfort due to RLS. English literature found on PubMed, Google Scholar, and Scopus was used in the present review. According to the titles and matters of the abstracts, each literature item connected to RLS was retrieved, analyzed, and reviewed. The 24 papers that were considered admissible were those that included manual approaches, exercises, and alternative RLS management, which were then analyzed for data by the authors. A consistent trend in the data demonstrated benefits in lessening RLS symptom severity across the 24 papers that met the selection criteria. Patients were chosen based on clinical diagnostic standards, and the effectiveness of stretching, exercise therapy, yoga, vibration therapy, reflexology and massage, muscular relaxation techniques, and electrical stimulation was determined. Our findings indicated that each type of therapy significantly affected the manifestations of the illness. Stretching, fitness training, and reflexology were very beneficial, with no side effects and shorter intervention periods.

## Introduction and background

Restless leg syndrome (RLS) is a common neurological disorder manifested by the urge to move the legs (rarely the arms) and unusual, unpleasant sensations (paraesthesia) deep in the legs. Movement frequently causes sensations to fade, reappearing during rest or inactivity, especially in the evening and at night. Paraesthesia that is unpleasant can seriously interrupt sleep [1]. RLS, also known as Willis-Ekbom illness, causes people to have an unavoidable drive to move their legs. RLS is a prevalent, chronic, multifactorial movement condition of the limbs. RLS is frequently linked to unusual, non-painful feelings that begin at rest and improve with movement. There is a daily trend where symptoms get worse at night. Each year, the United States has around three million instances [2]. It has been said that RLS is an idiopathic condition with no known etiology or that it is a symptomatic syndrome frequently linked to anemia, pregnancy, or last-stage renal failure. However, the condition is complex with underlying genetic, environmental, or both factors. Even though it is known that the onset can vary greatly, from an early age to over 80 years of life, the fact that the disease first manifests in childhood suggests a vital hereditary component. Despite prior underestimation of the condition's frequency, new epidemiological research using community samples has found that between 3% and 10% of people have the main RLS symptoms [3].

Pathophysiology of restless leg syndrome

Uncontrollable urges to move the legs are a hallmark of RLS, a neurological disorder typically accompanied by unpleasant sensations. Numerous processes have been proposed as probable causes of RLS, even though the exact pathophysiology of the disorder is not yet fully understood. RLS is known to be significantly influenced by brain iron levels. Blood-brain barrier (BBB) dysfunction has been associated with iron insufficiency in the brain, which can result in altered conduction of iron across the BBB and decreased iron storage in brain endothelial cells [4]. This deficit can cause oxidative damage and impact several functions, including the production and metabolism of neurotransmitters [5]. Due to their importance in regulating movement, dopaminergic cells are particularly vulnerable to iron deficiency, which can disrupt dopamine transmission and possibly cause symptoms of RLS [6]. RLS is closely linked with altered functioning of the dopaminergic system, which is vital in regulating movement and rewarding behavior [7]. It has been shown that drugs that alter dopamine levels can modify RLS symptoms. A lack of iron may cause dopaminergic dysfunction by changing how certain enzymes involved in dopamine production work. The midbrain's dopaminergic A11 cells, which seem to be involved in the synthesis of dopamine in the spinal cord, are considered crucial in the pathophysiology of RLS [8]. RLS is also linked to thalamic activity changes influenced by dopaminergic input [9].

Genetic factors greatly influence RLS susceptibility. Several genetic variations have been found to enhance the incidence of RLS, including MEIS1, BTBD9, PTPRD, and MAP2K5. These are examples of genes connected to the development of embryonic neurons, iron homeostasis, dopamine production, and sleep regulation. These genes may influence the development of RLS through their interactions with dopamine and iron metabolism pathways [10,11]. Studies on the pathogenesis of RLS have concentrated on brain areas such as the substantia nigra, dopaminergic A11 cell group, and basal ganglia. The spinal cord receives dopaminergic signals from A11 cells that are involved in the control of sensory, motor, and autonomic functions. RLS has been linked to thalamic and cerebral activity alterations and spinal cord hyperexcitability [12,13].

Clinical manifestation of RLS

Long documented, the RLS was first identified as an independent clinical entity by Ekbom in 1944. However, a sizable international study group has yet to agree on the parameters crucial for diagnosing this disorder. This group identified the following four RLS clinical features as the minimal criterion for diagnosis. Motor restlessness must also be present when patients feel compelled to move and use various motor techniques to relieve uncomfortable sensations associated with their restless legs. Symptoms must be more severe in the evening or at night or merely present at rest (i.e., lying down or sitting), with at least partial and severe movement of the affected limbs [14].

Diagnostic investigations

The clinical history is assessed to get the RLS diagnosis. Polysomnography should be performed if there is a medical potential for sleep apnea or if sleep problems continue even after RLS symptoms have been addressed. Given that RLS commonly occurs without anemia and may indicate iron deficiency, iron status (the serum ferritin level and iron saturation) should be assessed. Other laboratory examinations should be carried out [15]. Essential criteria for diagnosis of RLS [13,16,17]. According to the International Restless Legs Study Group, the criteria for RLS. Diagnosis includes a desire to move one's legs brought on by or stems from uncomfortable sensations through the legs up to the feet. When the urge to move the legs is present, it may or may not be accompanied by unpleasant sensations in the arms. The urge to move the legs may be present while sleeping or inactivity. The restlessness may or may not be entirely relieved by movement. The urge to move or unpleasant sensations may be worse at night than during the day or only occur at night.

Management of RLS

The main goal is to lessen or eliminate the uncomfortable feelings in the legs when the patient relaxes or sleeps. It is also essential to address RLS-related sleep disruptions. Managing daytime tiredness and fatigue brought on by sleep disruption will help patients feel rejuvenated. Improving the patient's well-being is the primary purpose. Both non-pharmacological and pharmaceutical treatments are used as RLS management techniques. Non-pharmacological Alternatives for managing RLS include modifying an individual's lifestyle and avoiding the consumption of aggravating substances like alcohol, coffee, and nicotine, indulging in exercises that stretch the muscles in the back of the legs, and applying heat through warm baths or hydrocollator packs, to relieve pain. Individuals with low serum ferritin levels may benefit from iron replacement treatment. In secondary RLS, treating coexisting diseases is crucial.

Pharmaceutical treatment includes dopaminergic agents. When non-pharmacological methods fall short, pharmacological agents are frequently utilized. Dopamine receptors are stimulated by dopamine agonists such as pramipexole, ropinirole, rotigotine, cabergoline, and pergolide. They are frequently used because they have fewer adverse effects than dopamine precursors. Clonazepam (Benzodiazepine): Although it helps enhance sleep and lessen arousals brought on by periodic limb movements in sleep (PLMS), it is not particularly successful in lowering the motor and sensory abnormalities connected to RLS. Opioids can lessen the incidence of PLMS and sleep disruptions, but they have misuse potential and undesirable side effects such as nausea and diarrhea. Anticonvulsants: Medications like gabapentin and pregabalin have proven helpful in treating the signs and symptoms of RLS and enhancing sleep. In particular, gabapentin enacarbil is well tolerated. Symptom-targeted drugs, lifestyle modifications, and diligent monitoring are all part of the multidimensional approach to managing RLS. Healthcare professionals need to tailor treatment regimens to each patient's unique demands and risk profiles [18].

## Review

Study selection

**Figure 1:**
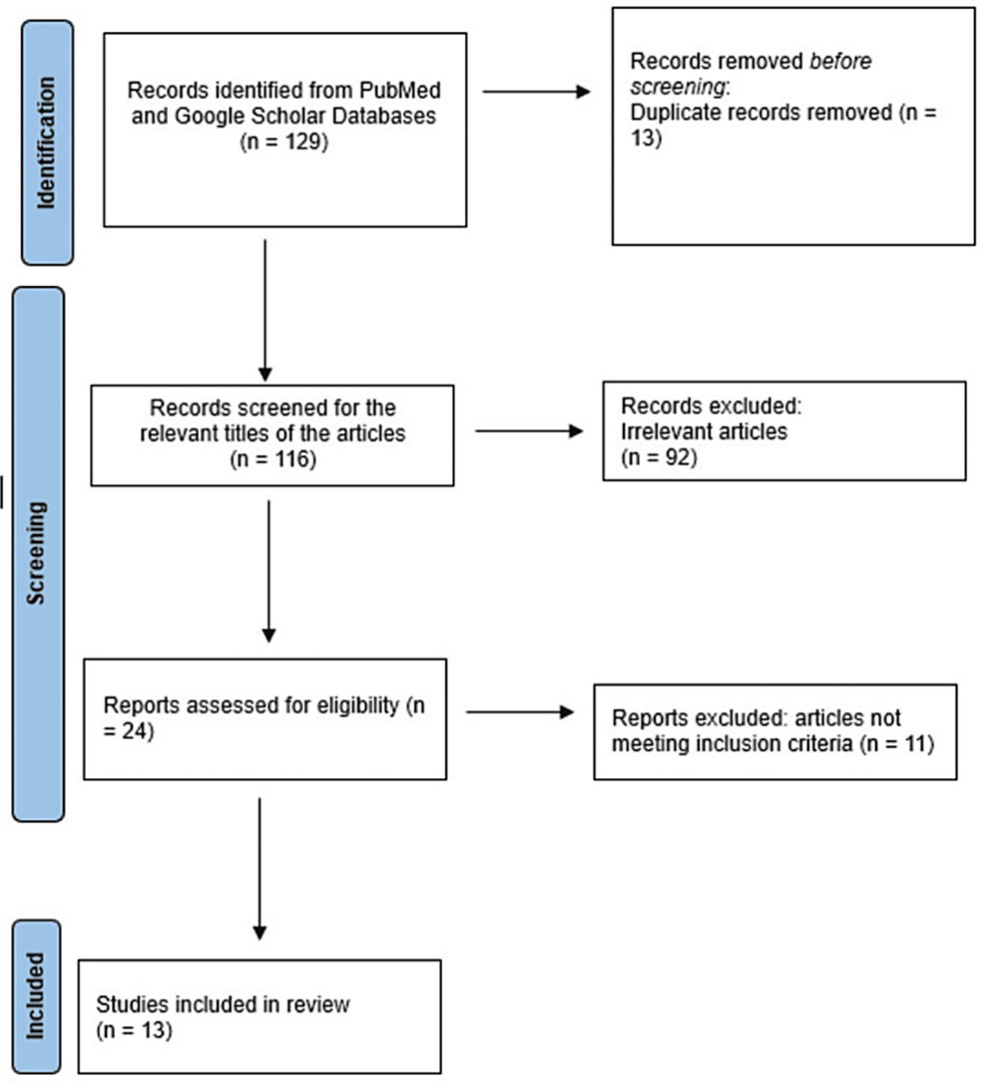
Identification of studies via databases and registers

A comprehensive literature search was conducted using various electronic databases, including Google Scholar and PubMed. The present study aims to investigate the impact of different physiotherapeutic interventions on the severity of RLS symptoms among suspected physiotherapy students. The search was performed from the year 2006 to the present date, using relevant keywords and phrases such as “restless leg syndrome,” “physiotherapeutic interventions,” “stretching exercises,” “resistance training,” and “quality of sleep.” During the literature search, 129 published articles were identified. Studies highlighting the impact of various approaches relieving RLS symptoms were included in the inclusion criteria for selecting the article. Among these, 24 papers were assessed and found relevant to the purpose of the study. Figure 1 represents the flow chart of the study selection and its database. 

The selected papers contain a wide range of topics related to RLS, including its definition, prevalence, diagnostic criteria, implications on sleep quality, and several treatment options for RLS symptoms. The usefulness of alternative therapies, such as yoga, reflexology, strength training, cryotherapy, and hot water thermotherapy, was also discussed in these papers. Research verifying evaluation methods and examining the socioeconomic impacts of RLS is also on the agenda. The chosen articles deepen our knowledge of the origins of RLS, how it impacts sleep, and the potential benefits of physiotherapeutic therapies for managing RLS symptoms. The research investigates the efficacy of strength training and stretching in treating RLS symptoms in physiotherapy students. The study's primary objective is to determine the prevalence of primary RLS among students, evaluate the efficacy of treatments, and assess the impact on sleep quality.

The outcomes of the current research may help physiotherapists, doctors, and educators develop effective treatment plans and treatments for persons with RLS, especially physiotherapy students who may be more vulnerable due to their study-related activities. The research results may also improve suggestions for treating RLS symptoms in various demographic groups based on evidence-based clinical practices. Table 1 provides details of articles selected for review.

**Table 1:** Summary of review of literature on restless legs syndrome RLS: Restless legs syndrome, PMR: Progressive muscle relaxation, ES: Electrical stimulation

Authors and year of publication	Study Type/ Methodology	Study Sample	Intervention	Results	Conclusion	Interpretation
Akbaş et al. (2023) [19]	Randomized controlled trial	A sample size of 52 was determined	progressive muscle relaxation method For eight weeks, participants practiced the skills thrice weekly.	The experimental group had considerably lower RLS intensity and Pittsburgh Sleep Quality Index (PSQI) ratings than the control group.	The findings suggested that using progressive muscle relaxation techniques during pregnancy can benefit RLS-suffering pregnant women by reducing their symptoms, enhancing their quality of life, and enhancing sleep.	Progressive muscle relaxation enhances sleep quality in pregnant women having RLS symptoms.
Mohammed Syam et al. (2022) [20]	An experimental study	examined 60 adult patients with end-stage renal failure	Twelve sessions of progressive muscle relaxation (PMR).	RLS severity was dramatically lessened by 38.89% in the sixth session and by 61.11% in the twelfth session.	The study recommends using progressive muscle relaxation in the therapeutic therapy of restless legs syndrome symptoms in maintenance hemodialysis patients since it suggests that it has a statistically significant favourable influence on RLS severity and many elements of well-being.	PMR results in reducing RLS, causing a sensation
Cho et al. (2022) [21]	Randomized controlled trial	46 individuals took part in the research. Participants were divided into two groups: an active group (n=22) and a false group (n=24).	A portable Electrical Stimulation(ES) device was used to give bilateral lower leg stimulation in the tapping mode (3 Hz),	Repeated measures ANOVA demonstrated that the active group considerably lessened the severity of their symptoms.	Both groups' symptoms improved during extended treatment, although the active group had more striking ES effects. In conclusion	ES therapy offers potential as a non-pharmacological treatment since it decreases symptoms, especially when the stimulation amount is accurate.
Innes et al. (2020) [22]	Randomized controlled trial	41 individuals participated, group 1(n=19) and group 2(n=22)	Group 1 followed the 12-week yoga sessions, and the group received an education and supportive fellowship (EF) programme for 30 mins each day	Both groups considerably improved after the intervention regarding RLS symptoms, severity, perceived stress, mood, and quality of life-mental health.	A significant decrease in RLS symptoms was also observed in the yoga group, and a probable dose-response relationship between homework practice and symptom alleviation was observed	yoga may be an effective RLS therapy, lowering symptoms, severity, and stress while enhancing mood and sleep quality
Azimpour et al. (2019) [23]	Randomized clinical trial	80 patients participated in the study. Each group has (n=40) participants.	Group 1 received vibration therapy, and Group 2 received massage therapy lasting 10 minutes each time, three times a week for a total of one month	Both vibration and massage therapy dramatically reduced the intensity of RLS symptoms and considerably increased the quality of sleep	The study found that both massage and vibration were effective in reducing the severity of RLS symptoms and enhancing the quality of sleep in hemodialysis patients, with vibration having a more pronounced effect than massage	the vibration was more effective than a massage at minimizing RLS symptoms and improving sleep quality.
Harrison et al. (2018) [24]	Randomized controlled trial	18 participants were assigned to two groups	Group 1: Trauma release exercises Group 2: Discussion group for six weeks	The study's findings, which showed that both trauma release exercises and discussion groups provided equivalent outcomes and improvements	Both interventions resulted in significant improvement in symptoms of illness.	trauma release exercises have a good impact in relieving RLS symptoms
Mansoor et al. (2016) [25]	randomized controlled trial	33 individuals were selected. A control group (n = 16) and an intervention group (n = 17) were formed from them	Intervention group: Half-hour leg stretching exercises three times a week.	After eight weeks of therapy, the intervention group showed a significant improvement in the intensity of restless leg syndrome symptoms compared to the control group.	This study highlights the therapeutic benefits of using stretching exercises in dialysis sessions to manage restless legs syndrome in hemodialysis patients.	Stretching exercise is effective in treating RLS in patients on hemodialysis.
Shahgholian et al. (2016) [26]	Randomized clinical trial	90 participants	Stretching for group 1 and reflexology for group 2 were practiced thrice weekly for four weeks.	stretching exercises and reflexology both significantly reduced RLS severity in comparison to the control group	reflexology and stretching exercises can both help to lessen the intensity of restless leg syndrome, making them recommended RLS treatments	Reflexology and stretching exercise plays an influential role in the management of RLS
Altunrende et al. (2014) [27]	A pilot study	19 participants	During each session of repetitive Transcranial Magnetic Stimuli(r TMS) train, 50 stimuli were given at 5 Hz with an intertrain interval of 50 s; 1,000 stimuli. (20 rTMS trains).	Individuals who underwent r TMS treatment reported improved scores on IRLS-RS.	After the tenth session, the IRLS-RS scores significantly improved due to the actual stimulation.	The tenth session's genuine stimulation considerably impacted the IRLS-RS ratings.
Giannaki et al. (2013) [28]	Randomized controlled trial	32 hemodialysis patients with restless legs syndrome	For a 6-month intervention, exercise training (n = 16), dopamine agonists (n = 8) or placebo (n = 8).	Exercise significantly reduced symptoms by 46% (p=0.001) and dopamine agonists by 54% (p=0.001). Training for physical activity significantly improved stamina, reduced muscle fat infiltration, and increased lean body mass. Dopamine agonists significantly improved sleep quality over exercise, and placebo suggests that both are effective in reducing RLS symptoms and depression in hemodialysis patients.	Dopamine had a higher effect on improving sleep quality than exercise training. However, both reduce symptoms of RLS.	Both interventions reduced depression scores but with potential benefits in physical measures and sleep quality.
Mitchell et al. (2011) [29]	Randomized controlled trial	34 participants	Over four weeks, the treatment group received twelve sessions of near-infrared light that lasted 30 minutes each.	The International RLS Rating Scale was used for assessment. The treatment group's RLS symptoms improved steadily for four weeks.	Infrared light therapy relieved discomfort due to RLS, and even four weeks posttreatment compared to baseline revealed a noticeably more significant improvement than the control group.	Near Infrared light treatment has the potential to reduce RLS severity and has a long-term effect.
Sakkas et al. (2008) [30]	A pilot study	14 participants	16-week aerobic exercise training programme on patients receiving hemodialysis (HD) who experience restless legs syndrome (RLS).	The International RLS study group rating scale score was significantly reduced by 42% in the exercise group after 16 weeks of supervised intradialytic aerobic exercise training, coupled with improvements in functional ability, exercise capacity, quality of life, and sleep quality.	This study emphasizes how exercise training may help HD patients with RLS manage their symptoms and improve their overall well-being	Aerobic exercises have a good impact on patients with RLS.
Aukerman et al. (2006) [31]	Randomized controlled trial	23 individuals. The exercise group (N = 11) and the control group (N = 12)	A conditioning routine consisting of lower-body resistance training and aerobic activity three days per week.	The exercise group engaged in The International RLS Study Group severity scale, and an ordinal measure of RLS severity was used to evaluate the symptoms of restless legs syndrome (RLS) at the beginning of the trial and at 3, 6, 9, and 12 weeks.	After 12 weeks, the exercise group (N = 11) had a substantially more significant reduction in symptoms compared to the control group (N = 12)	The prescribed exercise regimen dramatically reduced RLS symptoms during 12 weeks.

Discussion

In order to understand the effectiveness, comparative results, probable mechanisms, and clinical implications of a variety of physiotherapeutic therapies for restless legs syndrome (RLS), the current study carefully examined these interventions. The research under consideration covered a range of therapies, such as reflexology, electrical stimulation, stretching exercises, and workout plans. The analysis of these results offers an essential new understanding of the various approaches to treating RLS symptoms. Non-pharmacological therapies aim to cure RLS without using drugs and their side effects. It has been suggested that exercise can successfully cure RLS caused by idiopathic RLS [32]. The current review generates the efficacy of progressive muscle relaxation, strength training, aerobic exercises, reflexology, yoga, stretching, near infrared therapy, and electrical stimulation on patients on dialysis and pregnant females with RLS symptoms. Researchers recommend exercise training, transcutaneous spinal direct current stimulation (tsDCS), pneumatic compression devices (PCDs), light therapy, repetitive transcranial magnetic stimulation (rTMS), or acupuncture for the treatment of primary RLS, exercise training for the treatment of uremic RLS, and endovenous laser ablation (ELA) for the treatment of RLS with superficial venous insufficiency (SVI), based on the data that is now available. For primary RLS patients who struggle with sleeplessness, yoga, and PCDs should be considered. Yoga is said to considerably improve sleep and mood disruption without any negative consequences, making it a viable option for postmenopausal women with RLS. Additionally, PCDs represent a highly promising supplementary or alternative therapy for RLS since they are practical and appear to be successful in enhancing the intensity of RLS symptoms and all quality-of-life aspects [33]. Among all reviewed non-pharmacological and physiotherapeutic sources of intervention, yoga, aerobic exercises, PMR, and ES promise to provide significant relief from symptoms of RLS.

Although the reviewed studies offer valuable insights into the effects of physiotherapeutic interventions on RLS, several limitations warrant consideration, including small sample sizes and lack of long-term follow-up. Future research should employ larger sample sizes, standardized assessment tools, and more extended follow-up periods to provide more comprehensive evidence of the long-term benefits of these interventions. Research on non-pharmacological management of idiopathic and primary RLS among the younger generation is limited and is essential for future perspectives.

## Conclusions

The current research review outlines various therapy approaches that can be used to lessen RLS symptoms. It also considers the vital impact of different exercise regimens, stretches, and therapy modalities in alleviating pain and enhancing sleep quality in those with RLS caused by a primary illness or another underlying condition. This research highlights the potential of physiotherapeutic therapies as efficient management techniques for RLS symptoms. The trials under consideration collectively show the potential advantages of stretching routines, exercise plans, and electronic stimulation in reducing RLS-related suffering. A subtle approach to therapy selection is encouraged by the variety of processes at play and the possible superiority of electronic stimulation. Utilizing the knowledge gained from these therapies, doctors may improve the quality of life for those who suffer from RLS and optimize RLS management.
